# Genetic Basis of Metabolome Variation in Yeast

**DOI:** 10.1371/journal.pgen.1004142

**Published:** 2014-03-06

**Authors:** Jeffrey S. Breunig, Sean R. Hackett, Joshua D. Rabinowitz, Leonid Kruglyak

**Affiliations:** 1 Lewis-Sigler Institute for Integrative Genomics, Princeton University, Princeton, New Jersey, United States of America; 2 Department of Molecular Biology, Princeton University, Princeton, New Jersey, United States of America; 3 Graduate Program in Quantitative and Computational Biology, Princeton University, Princeton, New Jersey, United States of America; 4 Department of Chemistry, Princeton University, Princeton, New Jersey, United States of America; 5 Departments of Human Genetics and Biological Chemistry, David Geffen School of Medicine, UCLA, Los Angeles, California, United States of America; 6 Howard Hughes Medical Institute, UCLA, Los Angeles, California, United States of America; The University of North Carolina at Chapel Hill, United States of America

## Abstract

Metabolism, the conversion of nutrients into usable energy and biochemical building blocks, is an essential feature of all cells. The genetic factors responsible for inter-individual metabolic variability remain poorly understood. To investigate genetic causes of metabolome variation, we measured the concentrations of 74 metabolites across 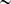 100 segregants from a *Saccharomyces cerevisiae* cross by liquid chromatography-tandem mass spectrometry. We found 52 quantitative trait loci for 34 metabolites. These included linkages due to overt changes in metabolic genes, e.g., linking pyrimidine intermediates to the deletion of *ura3*. They also included linkages not directly related to metabolic enzymes, such as those for five central carbon metabolites to *ira2*, a Ras/PKA pathway regulator, and for the metabolites, S-adenosyl-methionine and S-adenosyl-homocysteine to *slt2*, a MAP kinase involved in cell wall integrity. The variant of *ira2* that elevates metabolite levels also increases glucose uptake and ethanol secretion. These results highlight specific examples of genetic variability, including in genes without prior known metabolic regulatory function, that impact yeast metabolism.

## Author Summary

Many traits, from human height to *E. coli* growth rate, quantitatively vary across members of a species. Among the most medically and agriculturally important traits are levels of cellular metabolites, such as cholesterol levels in humans or starch in food crops. Metabolic variation in yeast also holds practical importance with some *Saccharomyces* strains better suited to making ethanol for biofuel and others tailored to making flavorful wine. This metabolic heterogeneity can be used to gain insight into general principles of metabolic regulation which effect metabolite abundance in eukaryotes. To this end, we examined inter-strain differences in metabolism in over 100 closely related *S. cerevisiae* strains. We identified over 50 genetic loci that control the levels of specific metabolites, including not only loci that encode metabolic enzymes, but also those that encode global cellular regulators. For example, differences in the sequence of *ira2*, an inhibitor of Ras, lead to differences in central carbon metabolite levels, and polymorphisms in *slt2*, a poorly characterized MAP kinase, alter levels of sulfur-containing metabolites. These findings provide insights into the mechanisms cells use to control metabolite concentrations.

## Introduction

Inter-individual differences in metabolism are of substantial biological importance. In humans, they underlie susceptibility to type II diabetes [1], obesity [2] and Crohn's disease [3], while in yeast, they contribute to the flavor profile of wine [4] and to the efficiency of ethanol generation [5], [6]. Accordingly, there has been growing interest in identifying the genetic loci responsible for inter-individual metabolome differences.

Over the past decade, the relationship between the metabolome and the genome has been increasingly studied, most thoroughly in the plant community [7]–[10]. Initial investigations followed metabolomic alterations in response to gene knockouts [8], [11]–[14], and this analysis has proven valuable for annotating gene functions [15]. Of late, decoding metabolic variation due to natural perturbations using quantitative genetics [16] has garnered increasing interest. Quantitative trait locus (QTL) studies have been performed on enzyme activities and metabolite concentrations in plants with greatest success for secondary metabolites [17]–[25]. Association of metabolite abundance variation with unsuspected genetic determinants has demonstrated the potential of metabolite QTL (mQTL) analysis for identifying genes with previously unknown enzymatic roles [17].

Metabolomic methods have been applied to determine how levels of metabolites are associated with gene segregation across intercrosses of mice, *A. thaliana* and yeast [26]–[28]. This has demonstrated that there is substantial genetic variation in primary and secondary metabolites, and this variation is governed by the segregation of relatively few mQTL hot spots [27], [28] whose epistatic interaction further shapes the metabolome [27]. These mQTL hot spots generally coincide with known eQTL hot spots, highlighting the extensive pleiotropy of these regions. While these studies have been able to associate regions of the genome with metabolic alterations, the residual unexplained heritability of these studies can be extensive, raising important questions about the power and reproducibility of QTL and mQTL analysis. Furthermore, the resolution of 100–200 F2 intercrosses is limited and identifying genetic associations has typically entailed identifying a locus of interest and reporting on the proximity to pathway-related enzymes, without searching rigorously for other linked genes that might play a regulatory role.

With the goal of discovering potential novel regulators of primary metabolism, we examined 74 metabolites involved in highly conserved core metabolic pathways of central carbon metabolism and nucleotide and amino acid biosynthesis. We found 52 significant linkages and experimentally verified the genes underlying three major linkage hot spots, including two linked genes responsible for altering S-adenosyl-methionine levels, neither with known metabolic roles. Additionally, we compared our metabolite results with the expression QTL results for the same cross [29] and discovered six overlapping hot spots. The largest mQTL hot spot is shared with the largest hot spot in the transcript data, and is caused by polymorphisms in a global regulator of cell signaling, *ira2*. Interestingly, while the expression QTLs linked to *ira2* were enriched for central metabolic enzymes, the variant of *ira2* that promoted high metabolite concentrations favored low enzyme transcript levels. This dichotomy can be explained because *ira2*-linked transcripts are primarily involved in oxidative metabolism, while linked metabolites are mainly associated with fermentation. These findings reveal the utility of mQTL analysis for identifying metabolic regulatory mechanisms.

## Results

To identify genetic loci responsible for inter-individual differences in the metabolome, we used a well-studied cross between a laboratory strain of yeast, BY4716, and a vineyard isolate, RM11-1a (hereafter referred to as BY and RM, respectively). These strains have both been sequenced, and they differ at 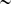 0.6% of base pairs [30]. Over 100 segregants from the cross have been densely genotyped and used in studies of the genetic basis of variation in protein and transcript levels [29]–[32] and a number of other phenotypes [33], [34].

Intracellular metabolites were harvested from yeast growing exponentially on aerobic, glucose-containing minimal medium by direct quenching and extraction in cold organic solvent [35]. The samples were then analyzed using two complementary targeted LC-MS/MS methods, one in positive ion mode and the other in negative ion mode [36]. Each method provides three-fold confirmation of metabolite identity based on parent ion mass, gas-phase fragmentation to a characteristic daughter ion, and LC retention time match to authenticated metabolite standard. We collected measurements from 13 independent replicates of the BY strain, 18 independent replicates of the RM strain, and two independent samples from each of 114 segregants. 105 compounds were reliably detected in at least one parent strain, and 79 of these were significantly different between the two strains at a false discovery rate (FDR) of 5% [37]. 74 of the 105 known compounds were measured in at least one-quarter of the segregants, and these 74 compounds were used for linkage analysis.

Many of these compound's levels show patterns of inheritance consistent with a complex underlying genetic basis. Based on the methods described for transcripts in Brem et. al. 2005 [32], we determined that 14 compounds showed transgressive segregation (the range in the segregants significantly exceeded that spanned by the parent strains) and 28 showed directional genetics (most segregants had levels intermediate between the parent strains). The observation of genetic complexity for most metabolite levels is concordant with what has been observed for other traits in this cross.

### Linkage analysis

We tested for linkage with R/qtl [38] and used permutations to establish that a LOD score of 3.4 corresponded to an empirical FDR of 10%. Of the 74 compounds tested, 34 showed at least one significant linkage (metabolite quantitative trait locus or mQTL; Table S1). The majority of these compounds (21 of 34) had one mQTL, 9 had two mQTLs, three had three mQTLs and one had four mQTLs, for a total of 52 detected mQTLs. Almost all the compounds for which mQTLs were detected differed significantly between the parental strains at an FDR of 5% (29 of 34). For 24 compounds that differed significantly between the parental strains, we did not detect mQTLs, most likely due to complex underlying genetics, with multiple loci of small effect. All compounds found to have significant mQTLs were primarily intracellular (as levels in biological samples were much greater than in media).

**Figure 1:**
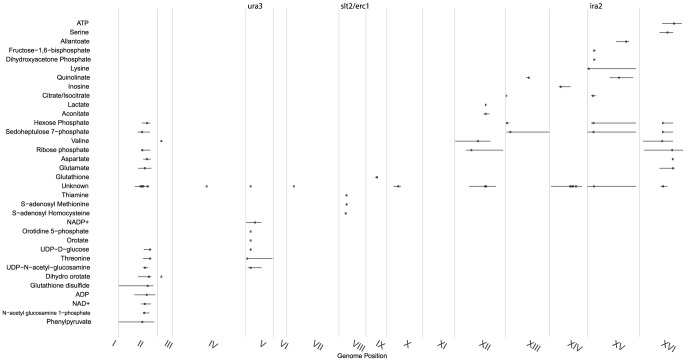
Distribution of significant linkages across the genome. Metabolite linkages that exceeded the 0.1 FDR significance threshold are plotted based on their most significant marker's genome location (indicated with a dot) with a 95% confidence interval. Continuous vertical lines represent chromosome ends. Numerals are placed at chromosomes' center. Genes investigated in this study are shown at top. mQTLs for ions of unknown identity were combined into a single class.

**Figure 2:**
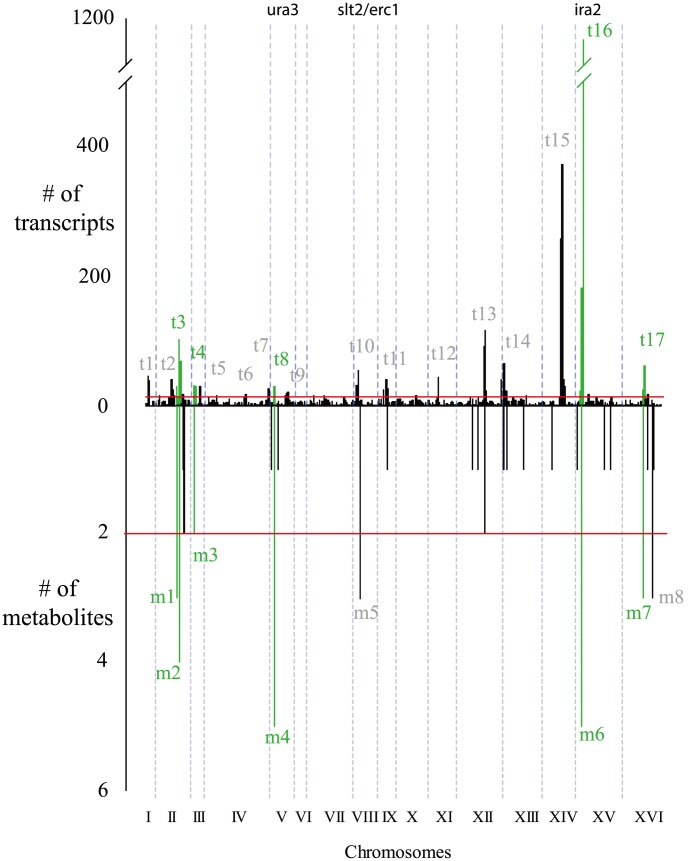
Similarities between metabolite and transcript linkage distributions. Significant linkages are binned in 10-eQTL hot spots are colored green. Dotted blue lines show chromosome ends. Red lines show the hot spot cutoff (see Methods for calculation).

The mQTLs were not uniformly distributed along the genome; rather, most fell within 8 “hot spots” with 3 or more compounds linking to each (Figures 1 and 2, Materials and Methods). To improve the power and thoroughness of this analysis (as well as a subsequent analysis of heritability and mQTL effect size), 42 ion peaks (20 mQTLs) with a defined 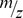 but unknown structural identity, were included. The observation of such hot spots, previously seen for other classes of traits, implies the presence of underlying polymorphisms with broad effects on the metabolome.

### Transcriptome and metabolome variation

We compared the metabolite linkage results with those for transcript abundance in the same cross [29]. Transcript linkages also cluster in hot spots, and the hot spots for metabolites and transcripts show a significant overlap in location, with six of eight metabolite hot spots also corresponding with transcript hot spots (p 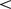 0.0001, based on permutation test) (Figure 2). Two metabolite hot spots did not have a corresponding eQTL hot spot: m8 on chromosome XVI (linked to levels of ribose-phosphate, aspartate and glutamate) and hot spot m5 on chromosome VIII (linked to levels of S-adenosyl-homocysteine, S-adenosyl-methionine, and thiamine). The absence of eQTL hot spots at these locations could be explained by underlying variants with effects on metabolism but not on transcript abundance, or by false negatives in the eQTL hot spot results, which could arise from variants with effects on only a few transcripts. Hot spot m5 is especially interesting since regulation of the methionine cycle is poorly understood in eukaryotes despite being implicated in cardiovascular disease [39], [40]. It will be discussed in greater depth below.

### Metabolic genes in confidence intervals

To determine whether changes in metabolites tend to be linked to genes with known roles in metabolism, we carried out functional enrichment analysis of genes located in mQTL confidence intervals. Genes were classified as “metabolic” based on inclusion in the iMM904 metabolism model [41]. The mQTL confidence intervals were found to be modestly but significantly enriched for metabolic genes. 471 out of a total of 904 metabolic genes in the yeast genome partially or completely overlapped with an mQTL 95% confidence interval. This is far greater than would be expected by chance, based upon permutation analysis (Figure S1; p 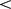 0.001). Each mQTL confidence interval was also examined specifically for the presence of metabolic genes in the same pathway as the linked metabolite (Table S2). Over half (31/52) of the confidence intervals were found to contain at least one metabolic gene from one of the pathways involving the linked metabolite.

### The *ura3* hot spot

**Figure 3:**
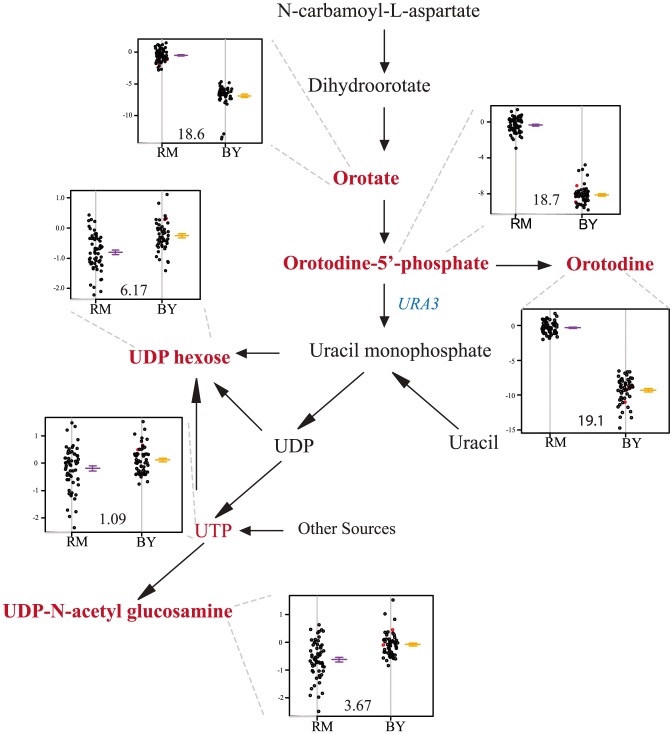
Levels of pyrimidine intermediates and products differ based on the *ura3* allele inherited. The relevant portions of the pathway are shown, with measured metabolites in red. The location of *ura3* in the pathway is shown in green. The accompanying plots show phenotype distribution of the segregants based only on the allele of *ura3* inherited: RM in purple, BY in orange. The *ura3* gene is defective in RM. All metabolite levels are log2(Segregant/RM). Compounds that were significantly linked to *ura3* locus (via LOD scores) are shown in bold.

Levels of five metabolites linked to a hot spot on chromosome V: orotate, orotidine, orotidine-5′-phosphate, UDP-D-glucose, and UDP-N-acetyl-glucosamine. All five are intermediates or products of pyrimidine biosynthesis (Figure 3). *Ura3*, a pyrimidine biosynthesis gene which carries an engineered deletion in the RM strain, is contained within the hot spot and lies within the 95% mQTL confidence intervals for all five compounds (Figure S2). Compounds upstream of *ura3* in the pathway show the greatest differences in abundance (as much as 128-fold), and particularly strong linkages (Figure 3). To confirm that this mQTL hot spot was governed by segregation of the engineered *ura3* deletion, *ura3* 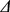, this RM allele was inserted into a BY background and metabolomic differences between BY and BY*ura3* 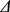 were assessed. Using a two-tailed t-test, two compounds were found to differ between these two conditions at a 0.05 FDR. These two compounds, orotate and orotidine-5′phosphate, are both associated with this mQTL hot spot; the deletion resulted in a 16 and 43-fold increase in their accumulation respectively. These results demonstrate that our approach can link changes in metabolite levels to a polymorphism (in this case, an engineered one) in a gene known to participate in the biosynthesis of the relevant metabolites.

### *Slt2* and *erc1* polymorphisms impact S-adenosyl-methionine levels

The mQTL hot spot on chromosome VIII (m5) is linked to levels of three metabolites: thiamine, S-adenosyl-methionine (SAM), and S-adenosyl-homocysteine (SAH) (Table S1). The overlap among 95% confidence intervals of the mQTLs for these compounds covers a region containing all or part of 14 genes (Figure S3). None of the genes in this region have a known connection with the sulfur-assimilation pathway. We identified *slt2* as a candidate for further evaluation due to the presence of a two amino acid indel polymorphism between BY and RM in a polyglutamine track; variation in the number of glutamines in this track has previously been implicated in stress response [42].

**Figure 4:**
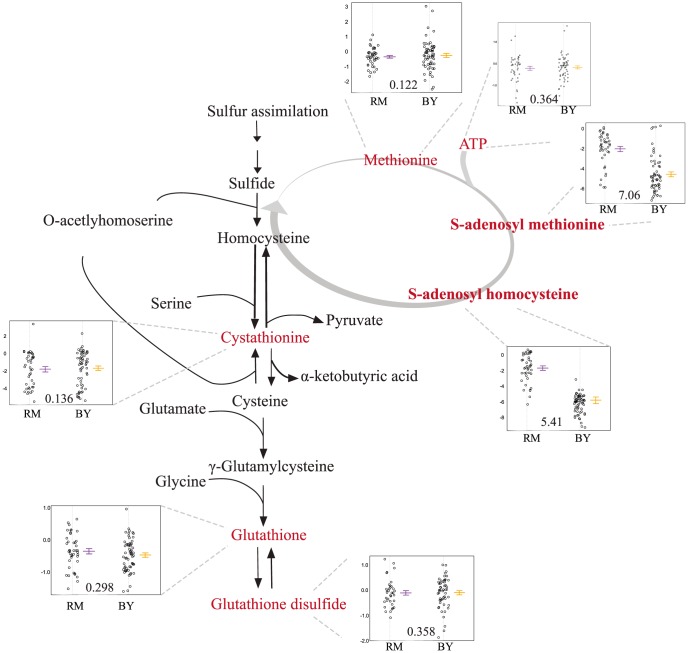
Levels of sulfur-assimilation intermediates differ based on the *slt2* allele inherited. The relevant portions of the pathway are shown, with measured metabolites in red. The accompanying plots display the phenotypic distribution of the segregants based only on the allele of *slt2* inherited: RM in purple, BY in orange. All metabolite levels are log2(Segregant/RM). Compounds that were significantly linked to *slt2/Erc1* locus (via LOD scores) are shown in bold.

**Figure 5:**
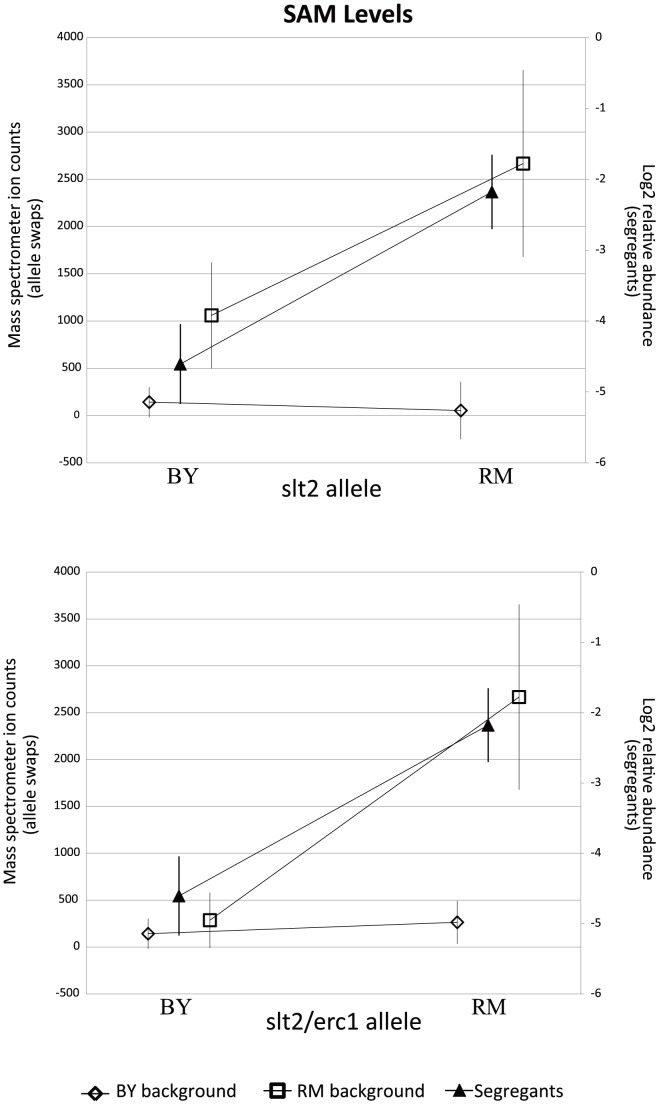
RM-inheriting segregants for *slt2* and *erc1* show significantly higher levels for SAM. Intensities (mean 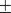 standard error) of SAM are plotted based upon the allele of *slt2* (top) and *slt2* and *erc1* (bottom). Mass spectrometer ion counts for BY background (diamonds) and RM background (squares) are shown on the left axis while segregants' log2 relative abundances (triangles) are indicated on the right axis.

Segregants inheriting the RM allele of *slt2* had significantly higher levels of SAM and SAH (Figure 4). To test the allelic effect of *slt2*, we created allele-replacement strains in both parental backgrounds and compared metabolite levels to those in the parent strains (Figure 5). In the BY background, the RM allele of *slt2* did not raise SAH levels above the limit of detection, nor did it result in a significant change for SAM (p  =  0.1598). However, in the RM background, the BY allele of *slt2* resulted in a three-fold decrease for both SAM and SAH (Figure 5; p 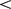 0.001). The difference in the effects of the allele swaps in the two backgrounds implies an interaction between the allelic status of *slt2* and other loci.

We considered the possibility that the effect of this locus is due to polymorphisms in multiple linked genes. We investigated a nearby gene, *erc1*, due to the presence of an indel polymorphism that causes a frameshift which alters 37 residues and extends the peptide by 43 amino acids in the RM background. *Erc1* has also been shown to have an effect on SAM levels when overexpressed in saké strains of S. cerevisiae [43]–[45]. *Erc1* is located 3 kb (approximately 1 cM) from *slt2*, and thus the alleles of the two genes segregate together as a haplotype. We used the *slt2* allele replacement strains to create strains in which both genes were replaced with the alternative alleles. In the BY background, replacing both *slt2* and *erc1* with the RM alleles led to a significant increase in SAM (p  =  0.019) compared to the original BY strain, but the level of SAM was still much lower than in RM (Figure 5). In the RM background, replacing both genes with the BY alleles led to significantly lower levels of both metabolites compared to either the original RM strain or to the *slt2* replacement alone (p 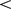 0.001 for all comparisons). These results suggest that polymorphisms in both *slt2* and *erc1* alter the levels of SAM-cycle compounds in these strains, with other undetected loci also playing a role in the observed variation.

### *Ira2* polymorphisms alter central metabolites

A mQTL hot spot on chromosome XV (m6) is linked to five central carbon metabolites: glucose-6-phosphate (G6P) and its isomers (which were not distinguished by the LC-MS method used here), fructose-1,6-bisphosphate (FBP), sedoheptulose 7-phosphate (S7P), dihydroxyacetone phosphate (DHAP), and (iso)citrate. The overlap among the 95% confidence intervals of the mQTL for each compound covers a region containing all or part of 13 genes (Figure S4). We focused on *ira2* as a candidate gene because it has a known function as a regulator of the Ras/PKA pathway [46], a known effector of glycolytic flux [47], and because we previously showed that polymorphisms in *ira2* underlie a major eQTL hot spot (t16) at the same locus in this cross [29], [48]. *Ira2* is a Ras-related GTPase [46], [49], [50], with *ira2*-catalyzed GTP hydrolysis leading to inactivation of Ras. The eQTL expression patterns suggested that *ira2* is hypoactive in the BY strain.

**Figure 6:**
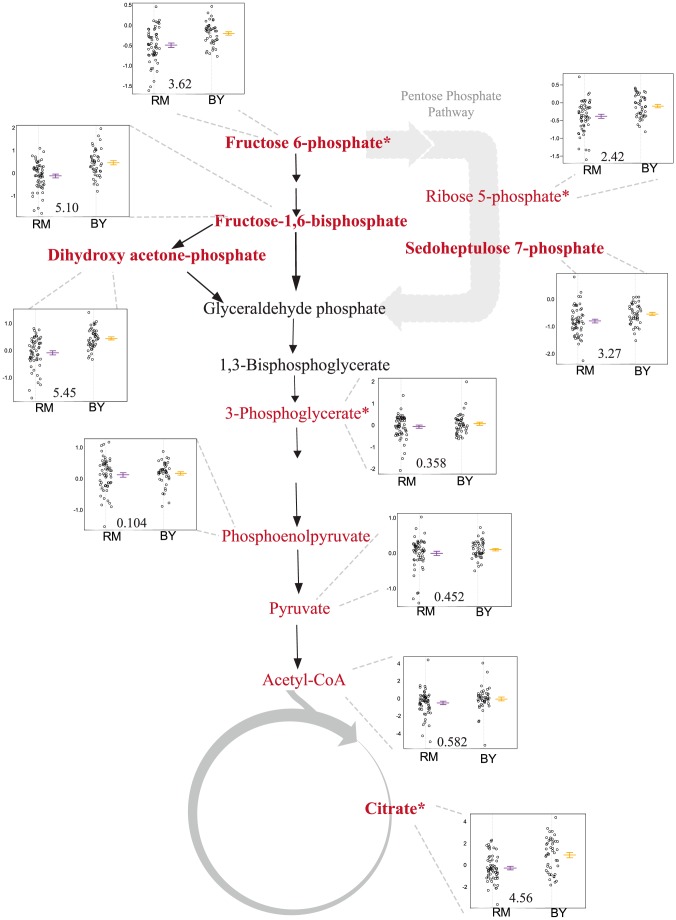
Levels of glycolysis, pentose phosphate pathway and TCA intermediates differ based on the *ira2* allele inherited. The relevant portions of the pathway are shown, with measured metabolites in red and significant linkages shown in bold. The accompanying plots show phenotype distribution of the segregants based only on the allele of IRA2 inherited: RM in purple, BY in orange. All metabolite levels are log2(Segregant/RM). LOD score for the closest marker is also shown. *includes analytically indistinguishable isomers.

**Figure 7:**
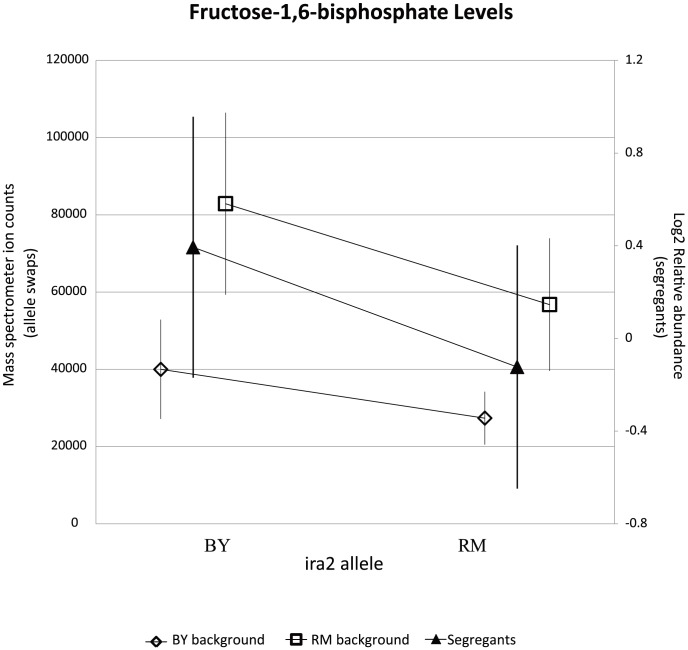
RM-inheriting segregants for *ira2* show significantly lower levels for fructose-1,6-bisphosphate. Intensities (mean 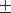 standard error) of FBP are plotted based upon the allele of *ira2*. Mass spectrometer ion counts for BY background (diamonds) and RM background (squares) are shown on the left axis while segregants' log2 relative abundances (triangles) are indicated on the right axis.

Segregants that inherit the BY allele of *ira2* showed higher levels of all five linked metabolites than those that inherit the RM allele (Figure 6). To test the allelic effect of *ira2*, we compared metabolite levels of *ira2* allele-replacement strains in both backgrounds [29] to the original parent strains (for FBP, see Figure 7; for other metabolites, see Figure S5). In the RM background, the BY allele of *ira2* led to significantly higher levels of three compounds (p 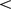 0.01 for sedoheptulose-7-phosphate, FBP, DHAP). In the BY background, the RM allele of *ira2* led to significantly lower levels of all five metabolites (p 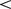 0.05). These results demonstrate that polymorphisms in *ira2* contribute to the observed variation in these five central metabolites.

Metabolites can accumulate due to either increased production or decreased consumption. To distinguish whether the BY allele of *ira2* was enhancing central carbon metabolic flux versus inhibiting metabolite consumption, we analyzed glucose uptake in the BY and RM parent strains, as well as in *ira2* allele-replacement strains in both backgrounds. Glucose uptake rate did not differ significantly between the two parental strains. In the two allele-replacement strains, however, glucose uptake diverged markedly. In the RM background, the BY allele of *ira2* led to 45% faster glucose uptake, whereas in the BY background, the RM allele led to a 20% decrease (Figure 7). The main fermentative product of glucose is ethanol, so the rate of ethanol excretion in *ira2* allele-swap strains was measured using 1H NMR. In either background, the BY allele of *ira2* led to a significant increases in ethanol excretion (p 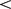 0.05). These results demonstrate that polymorphisms in *ira2* control central carbon metabolic flux, with the BY allele inducing both higher metabolite levels and fluxes. In the parental strains, the metabolic flux impact of the *ira2* polymorphism is presumably offset by differences at other loci.

Because polymorphisms in *ira2* result in differences in expression of 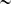
1300 genes [29], we considered whether expression differences in central carbon metabolism genes might underlie the observed metabolic changes. Of 70 known central carbon metabolism genes (i.e., those with roles in glycolysis, pentose phosphate pathway, citric acid cycle, and oxidative phosphorylation from yeastgenome.org), 32 genes' expression linked to the *ira2* locus in glucose media (Table S3). This significantly exceeds the number of linkages expected for a random set of genes (p 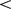 0.01, Fischer's exact test). Remarkably, of the 32 linked genes, 28 are less highly expressed in the BY strain, which has higher levels of G6P, FBP, S7P, DHAP, and (iso)citrate. Thus, paradoxically, the BY allele of *ira2* promotes higher central carbon metabolite levels while repressing central carbon metabolism gene expression.

Insight into this paradox is provided by the nature of the regulated genes: 28 of the 32 central carbon metabolism genes that link to *ira2* tend to be more highly expressed in ethanol than in glucose [29]; i.e., the primary transcriptional regulatory role of *ira2* seems to be in enhancing expression of genes required for respiratory growth. In contrast, with the exception of (iso)citrate, the linked metabolites are indicative of active fermentation. The accumulation of (iso)citrate in the BY strain is consistent with the lower expression of the primary isocitrate consuming enzyme (*idh1*) from the BY allele of *ira2*. Taken together with the data showing that the BY allele of *ira2* promotes glucose fermentation, one obtains a coherent view: *ira2* activity is lower in the BY strain. This leads to decreased expression of genes required for respiration, more need for fermentative ATP production, and higher levels of the glycolytic intermediates G6P, FBP, and DHAP.

### Heritability of metabolite levels

**Figure 8:**
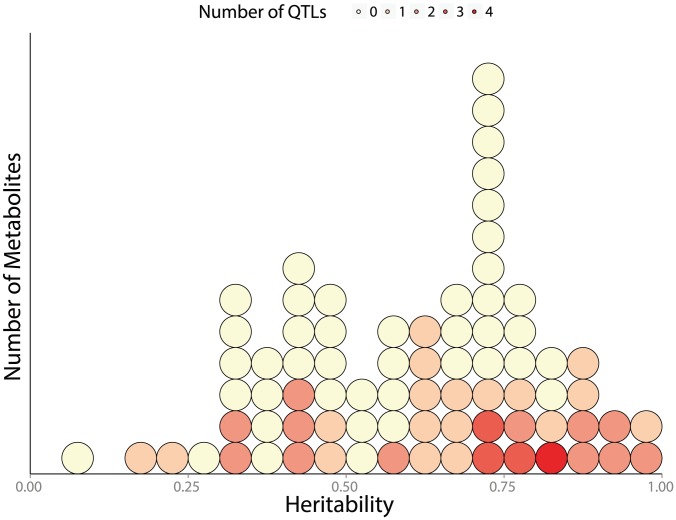
Distribution of broad sense heritability ( 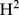
 **) across measured metabolites.** each circle represents a single metabolite, colored according to how many QTLs are associated with its abundance. 114 metabolites are shown: 74 known metabolites with 52 detected mQTL and 42 unknown metabolites (with known m/z, but unknown identity) associated with 20 additional mQTLs.

**Figure 9:**
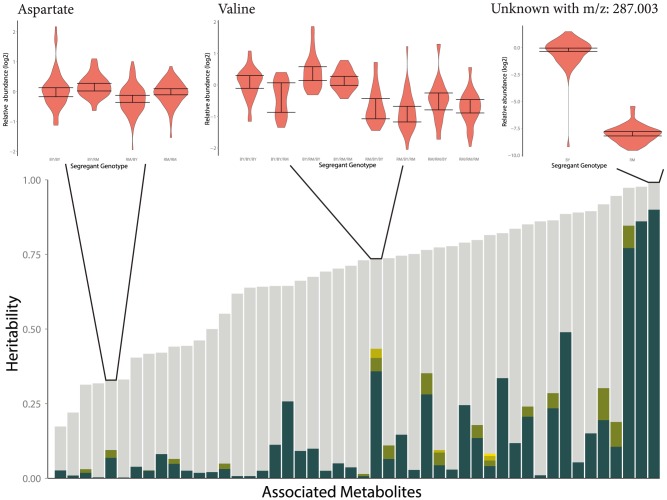
Fraction of broad-sense heritability explained by identified mQTLs. Each stacked bar represents a single metabolite which was significantly associated with at least one locus. The height of the bar is the broad-sense heritability of the metabolite's abundance, and the coloration partitions this heritability into unexplained heritability (gray), and the effects of each mapped QTL (colors). Three examples are given to demonstrate the variable effect sizes observed across metabolites. The distribution of metabolite abundances for a genotype is shown as a violin plot, and a 95% confidence interval for the median of each genotype is reported with error bars. This confidence interval was determined using a percentile bootstrapping method [73].

We can only relate metabolite abundance variation to genetic heterogeneity across segregants when there is substantial genetic variation affecting metabolite levels in the first place. Previous estimates of broad-sense heritability [51] in *A. thaliana* have suggested moderate heritability of metabolite traits across globally-distributed strains [20], while segregants showed substantially lower heritability of metabolite traits than expression traits (an average of 25% and 65% respectively) [27], [52]. We found extensive heritable variation of metabolite abundance in this study, with an average broad-sense heritability of 62%. This indicates that there are likely larger metabolic differences segregating between BY & RM than within the Bay 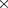 Sha *A. thaliana* cross. Greater levels of heritability across metabolites are associated with an increased number of detected mQTLs (p  =  0.014); this is evident in Figure 8, which shows linkage numbers as a function of heritability. The effects of these QTLs can be seen by determining the fraction of the variance in metabolite abundance that is explained using QTL genotypes (Figure 9). Effect sizes and the total fraction of heritability explained vary greatly across metabolites, with some mQTLs explaining the vast majority of genetic variation, others collectively explaining a sizable portion through the joint additive effects of multiple loci and others still explaining little of the total variance. The large fraction of unexplained metabolite abundance heritability could be owing to two factors: insufficient power to detect multiple loci of small effect, or the non-additive interaction between loci [27], [53].

## Discussion

We used high-throughput metabolite phenotyping in a cross of two divergent strains of yeast to find 52 linkages for 34 metabolites. We have detected linkages for a majority of compounds with significant differences between parental strains, as well as for a few compounds without such differences. Many metabolites show transgressive segregation, with levels in progeny strains outside the range of the parents; the parental strains likely carry alleles with opposing effects, with some segregants that inherit combinations of alleles that result in extreme metabolite levels, as has been observed for transcript levels [32]. Such opposing effects in the parent strains were also evident in control of glycolytic flux, which is similar in the parental strains, but diverges upon an *ira2* allele swap.

*Ira2* is a regulator of cell signaling, not metabolism per se. Nevertheless, allelic differences in *ira2* have a broad impact on central carbon metabolism at the level of transcripts, metabolites and flux. The hypoactive variant of *ira2* found in the BY strain is associated with decreased expression of oxidative metabolism transcripts, higher levels of citrate, glycolytic intermediates, and sedoheptulose-7-phosphate, as well as higher glycolytic flux. These observations are consistent with active *ira2* inducing oxidative metabolic genes, which in turn decrease the glycolytic flux required to fulfill ATP production. This raises the intriguing possibility that, due to the efficiency of oxidative ATP production, the extent of residual oxidative phosphorylation during yeast fermentative growth is a major determinant of glycolytic flux. More direct inhibition of glycolysis by the BY variant of *ira2*, e.g., through inhibition of phosphofructokinase-2, is also a possibility.

Perhaps the most exciting use of yeast mQTL mapping is to discover novel regulators of metabolism. In this respect, we have found linkages between levels of SAM and SAH and two proteins, *slt2* and *erc1*, with no previously known metabolic regulatory role. These two proteins interestingly segregate as a complex haplotype. SAM and SAH are key metabolites from the perspective of epigenetics; they are substrates and products, respectively, in DNA and histone methylation. Through epigenetics or other mechanisms, SAM and SAH may impact a broad range of diseases, e.g., of the cardiovascular system [39], [40], liver [54], or brain [55]–[57]. *Slt2* is part of a MAP kinase cascade responsible for maintaining cell wall integrity, and thus contributing to fitness during osmotic stress. *Erc1* was identified for conferring ethionine resistance [42]–[45], [58]–[60]. While SAM and SAH (as well as a thiamine, which also links to the same locus), are notable for containing sulfur, neither *slt2* nor *erc1* is regulated transcriptionally in response to sulfur availability [61], [62]. Both sulfur metabolites and *slt2* have been associated with the cell cycle (in the case of *slt2*, via the cell cycle transcription factors *swi4* and *swi6*) [63]–[67]. The molecular mechanism by which *slt2* and *erc1* polymorphisms regulate SAM and SAH levels remains, however, to be elucidated. The discovery of the underlying mechanisms, may in turn, inform the overall interplay between metabolism, epigenetics, and the cycle cell. Thus, mQTL analysis provides a powerful tool for integrative systems biology.

The BY 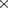 RM cross utilized in this work has been previously used to characterize metabolite abundance variation with quantitative NMR in Zhu et al. 2012 [28]. While the designs of these studies are very similar, the use of LC-MS in our study, as well as different experimental procedures, resulted in substantial differences in the observed mQTL hot spots, allowing us to expand upon and provide an alternative explanation for the basis of some of these controlling regions. Of the 56 metabolites reported in our study, 27 were also quantified in Zhu et al., and of the 16 metabolites for which Zhu detected significant linkage, 12 were shared between the two studies.

Three hot spots are shared between these two studies: those which we have shown are due to variation in *ura3*, *slt2*/*erc1*, and *ira2*. In Zhu et al., the *ura3* auxotrophy was implicated through its linkage with orotate and dihydroorotate elevation; we have confirmed these effects both statistically and through direct experimental manipulation of *ura3*, and also expanded them to other metabolites in the pathway. In both studies, SAM and SAH were linked to the *slt2*/*erc1* locus, but Zhu et al. did not discuss this hot spot, and they did not identify or propose underlying genes. Zhu et al. also mapped the abundance of glycerol, lysine, tyrosine and trehalose to the region containing *ira2* and *pmh7*. They concluded that variation in *pmh7* was the causal source of these metabolic alterations, but this conclusion was based on a weak knockout phenotype, rather than on an allele replacement. Of these metabolites, we were only able to quantify lysine, which was not linked to this region in our study. It is therefore difficult to determine whether *ira2* and *phm7* function as a complex locus, similar to *slt2/erc1*, with both genes playing a role in variation of the same or different sets of metabolites, or whether *ira2* is the only gene in the region that influences metabolite variation.

The remaining mQTL hot spots of Zhu et al. were associated with amino acid metabolism and were not observed in our study, perhaps because of differences in growth conditions: synthetic compete medium in Zhu et al. vs. supplemented minimal medium in this study. Such mQTL hot spot dependence on growth conditions would be analogous to gene-environment interaction eQTLs (gxeQTL) previously identified in the BY 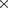 RM cross [29]. This observation suggests that mQTL analysis under a variety of growth conditions could be an important method for discovering novel metabolic regulatory mechanisms.

## Materials and Methods

### Culture conditions

We used strains generated from the cross between BY4716 (MAT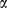 *lys2* 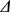) and RM11-1a (MATa *leu2* 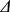 *ura3* 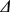); these strains have been extensively studied for a variety of quantitative phenotypes [29]–[34], [68]. Growth medium was comprised of 6.7 g/L Yeast Nitrogen Base (YNB) without amino acids, 2% (w/v) glucose as the sole carbon source, and was supplemented with leucine, lysine and uracil (final concentrations 100 mg/L, 30 mg/L, 20 mg/L respectively) to complement the strain auxotrophies. Yeast were grown in this medium using a filter culture technique that enables rapid sampling of metabolism without perturbation of the cultured cells [35]. In brief, strains were grown aerobically in liquid minimal medium to an 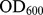 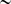 0.1, at which point 5 mL of the culture was transferred by filtration to the surface of an 82 mm, 0.45 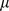
m pore size nylon membrane, which was subsequently placed atop a medium-loaded agarose plate as described in Brauer et al. [35]. The filter cultures were grown aerobically to mid-log phase (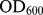 in 5 mL wash  =  0.2–0.6, for 3–5 hr, approximately 2–4 doublings) before metabolism quenching and metabolome extraction. All growth was at 30
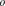
C. Cultures were grown in triplicate, with two filters used for metabolite extraction and the third filter for OD measurement.

### Metabolite extraction

The cell-loaded filter membrane was quenched by placing it cell-side down in 2 mL of acetonitrile/methanol/water (40∶40∶20) at 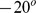
C. After 15 min, residual cells were rinsed off of the filter and the 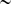 2 mL cell-extraction solvent mixture was centrifuged at 13,200 rpm for 5 minutes at 4°C to generate a clear supernatant. 90
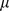
L of this clear metabolome extract was mixed with 10
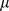
L of a mixture of isotope-labeled internal standards to yield an analysis-ready sample. Samples were stored at 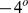
C until analysis, which was completed within 24 h of sample generation.

### Metabolome quantitation and pre-analysis

Two different LC separations were coupled by electrospray ionization (ESI) to Thermo TSQ Quantum triple quadrupole mass spectrometers operating in multiple reaction monitoring (MRM) mode. Positive-mode ESI was coupled to hydrophilic interaction chromatography (HILIC) on an aminopropyl column; negative-mode ESI was coupled to reversed-phase chromatography with an amine-based ion pairing agent [69], [70].

Raw LC-MS/MS data from both runs were analyzed using the MAVEN software [71]. The results of this automated analysis were manually verified in all cases. Peak quantitation was based on the average of the top three points in the peak.

For linkage analysis, compounds detected in fewer than 25% of samples were discarded; for the remaining compounds, when signal was not detectable, raw ion counts were floored to 32, which is approximately the lower limit of detection. Duplicate samples of the same strain were averaged and then divided by the associated OD at extraction to normalize for any sample-size differences.

Each day the RM11-1a strain was also run under this method. To correct for inter-day variance in raw signal intensities, log-ratios between segregant and the same-day RM values were used for each compound.

### Analysis of metabolome differences between the parental strains

For each compound's abundance data, an ANOVA of the form phenotype 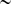 strain was performed in R using the aov function to compute p-values. These p-values were then false-discovery-rate corrected to assess statistical significance. Tests for mode of inheritance were conducted according to the formulae laid out in Brem & Kruglyak [32].

### Media extraction

To determine which metabolites may appear abundant by virtue of the extraction procedure, we compared metabolite levels from mock extracted cells to the parental strains using a one-tailed t-test and we found six compounds at levels comparable to biological samples. Four of these metabolites were included in the media as vitamins or supplements: leucine/isoleucine, nicotinate (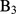), pantothenate (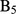), and 4-Pyridoxic acid (a 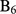 catabolite). Two additional metabolites had elevated levels that likely resulted from systematic contamination: deoxyribose-phosphate and D-glucono-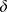-lactone-6-phosphate. No QTLs were associated with any of these compounds, so their inclusion should not impact our subsequent analysis.

### Segregant linkage analysis

We used genotypes at 2,820 SNP markers that were previously genotyped in individual segregants [32], giving an average spacing between markers of 4.3 kb or 1.5 cM. With over 100 segregants, we would expect to see an average of more than one recombination event between adjacent marker pairs in this cross. Linkage analysis was performed using the qtl package in R [38]. We used the normal model and nonparametric method, assessing significance through the built-in permutation test. We computed 100 permutations of the qtl profile for every metabolite; linkage scores that were in the top 10% of this set were considered significant. This cutoff differs for each metabolite, ranging from a LOD score of 3.14 to 3.58 with an average of 3.35. We calculated confidence intervals using the bayesint function with a probabilit y of 0.95. This is generally considered more conservative than intervals calculated based on a 1.5 LOD drop; secondary peaks on the same chromosome will result in larger intervals.

### Allele replacement strains

The allele replacement strains for IRA2, SLT2, and ERC1 were constructed according to methods laid out in Gray et al. [72] and Smith et al. [29]. The strains used were BY4724 (MATa LYS2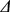 URA3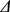), BY4724 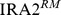, BY 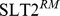, BY 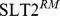 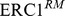, ACY753 (an RM MATa URA3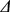), and RM 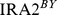, RM 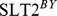, RM 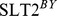 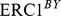. Allele swap strains were compared to their parental strain using paired t-tests.

### Identification of metabolic genes in confidence intervals

Confidence intervals for each QTL were computed as described above. Using the intervals package in R and the position and name of metabolic genes from Mo et al. [41], we created a dataset of all metabolic genes in the *S. cerevisiae* genome. The intervals_overlap() command returned how many and which metabolic genes fully or partially overlaped with our confidence intervals. To compute significance for all confidence intervals, we randomly permuted the position of the intervals 10,000 times, each time recording the total number of metabolic genes contained in the intervals.

To look at pathway-specific metabolic genes for each metabolite, we compared the SGD list of genes in all pathways for that metabolite with the list of all metabolic genes in that metabolite's confidence interval (pathway information was downloaded from Yeast Biochemical Database available at *Saccharomyces* gene database http://www.yeastgenome.org/biocyc on 29 September 2009). For metabolites with multiple linkages, each confidence interval was examined separately.

### Comparison between metabolite and transcript datasets

All transcript data was taken from Smith and Kruglyak [29], using only the data for glucose-grown cells.

For comparing linkage location, the genome was broken into 10 kb bins and the peak of each linkage (transcript and metabolite) was assigned to a bin. A bin was considered to have an excess of linkages if the number exceeded the number expected by chance by Poisson distribution. Given the number of metabolite-linkages (52) and bins (1216) we have 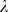  =  0.0428, and we used a Bonferroni corrected significance (p 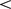 4.11*10-5); this resulted in significance for any bin that linked to three or more metabolites. For transcript-linkages 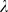  =  4.151 and the significant hot spots are defined by have 14 or more linkages. Hot spots in immediately adjacent bins were accepted as part of the same hot spot. When comparing hot spots between the datasets, they were considered shared only if they inhabited the same linkage bin.

### Heritability and study reproducibility

For each metabolite, segregants with two quantifiable biological replicates were isolated and the variance within replicates was compared to the total across all samples. This is effectively subtracting the environmental variance from the total phenotypic variance to yield the genetic variance. The ratio of genetic variance to phenotypic variance is the broad sense heritability (equation 1)
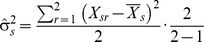


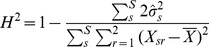
(1)


The association between the number of QTLs found for a metabolite and the metabolite's heritability was found by modeling the number of detected QTLs as an approximately poisson trait and predicting this value using poisson regression.

## Supporting Information

Figure S1 Accumulation of metabolic genes in random distributions of intervals. Confidence intervals were randomly permuted across the yeast genome. All genes classified as metabolic that overlapped with a confidence interval were counted (see Materials and Methods). This was repeated 10,000 times and the distribution seen is shown. The red arrow shows were the actual count is relative to the distribution (471), where only 0.7% of permuted values were greater.
(EPS)

Figure S2 95% Confidence Intervals of Chromosome V-linked metabolites. 95% Confidence intervals were computed using the bayesint function in R/QTL. Shown in black is the interval, the red marks are the location of the specific marker with the highest LOD score for the respective metabolite. In blue are the ORFs of local genes. URA3 is the closest gene to all five markers and within all five intervals.
(EPS)

Figure S3 95% Confidence Intervals of Chromosome VIII-linked metabolites. 95% Confidence intervals were computed using the bayesint function in R/QTL. Shown in black is the interval, the red marks are the location of the specific marker with the highest LOD score for the respective metabolite. In blue are the ORFs of local genes.
(EPS)

Figure S4 95% Confidence Intervals of Chromosome XV-linked metabolites. 95% Confidence intervals were computed using the bayesint function in R/QTL. Shown in black is the interval, the red marks are the location of the specific marker with the highest LOD score for the respective metabolite. In blue are the ORFs of local genes. IRA2 is within all five intervals.
(EPS)

Figure S5Impact of IRA2 allele on glycolysis. Segregants inheriting the RM allele of IRA2 show significantly lower citrate, dihydroxyacetone phosphate, hexose phosphate and sedoheptulose 7-phosphate levels. Relative metabolite concentrations (mean 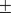 standard deviation) are plotted based upon the allele of IRA2. Absolute ion counts for BY background (diamonds) and RM background (squares) are plotted on the left axis while segregants (triangles) relative intensities are plotted on the right axis.(EPS)

Table S1Metabolites and their linkage LOD-scores. All 52 linkages are listed, sorted by metabolite name. Metabolites with multiple linkages are sorted by LOD-score. The chromosome and position of the closest marker are also given. For metabolites detected in both parental strains, the p-value of metabolite level differences between the parents is also shown. FDR of 5% corresponds to a p-value of 0.0898. * Same compound but in different ionization modes. 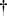 considered same compound.(PDF)

Table S2Examining confidence intervals for pathway genes. Compounds are shown with the number of pathway genes and metabolic genes captured in their confidence intervals. Pathway genes for each compound are specified in the third column. For compounds with multiple linkages, metabolic gene number and pathway gene names are broken down by the chromosome of the linkage. Glutathione and glutathione-disulfide are combined, as are the positive and negative mode measurements for S-adenosyl-homocysteine. * While alcohol dehydrogenase (ADH1) is not specified as a gene in the same pathway as these metabolites, it is mentioned due to its role in glycolysis. 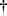 Same as S-adenosyl-L-homocysteine-nega-1.(PDF)

Table S3 eQTLs containing IRA2 from carbon cycle genes. eQTLs were taken from Smith et al. [29] for genes affiliated with the carbon cycle, as determined from www.yeastgenome.org. Genes with eQTLs containing IRA2 are marked whether the eQTL was detected in media containing either ethanol or glucose as a carbon source. Each genes average expression levels were also compared dependent on the allele of IRA2 and noted.
(XLSX)
