# Supplementary material for: Genetic Basis of Metabolome Variation in Yeast
Source: PLoS Genet. 2014 Mar 6;10(3):e1004142. doi: 10.1371/journal.pgen.1004142 (PMC3945093; doi:10.1371/journal.pgen.1004142)
Supplement: Table S1 — Metabolites and their linkage LOD-scores. All 52 linkages are listed, sorted by metabolite name. Metabolites with multiple linkages are sorted by LOD-score. The chromosome and position of the closest marker are also given. For metabolites detected in both parental strains, the p-value of metabolite level differences between the parents is also shown. FDR of 5% corresponds to a p-value of 0.0898. * Same compound but in different ionization modes. considered same compound. (PDF) [file pgen.1004142.s006.pdf]

Name	PubChem ID	LOD score	Chromosome	Position (cM)	Parent's P-value
aconitate	643757	6.032	12	315.18	0.149694583
ADP	6022	3.650	2	227.24	0.0000781
allantoate	5287444	3.838	15	334.51	N/A
aspartate	5960	4.681	2	227.24	0.00000024
aspartate	5960	4.633	16	268.52	0.00000024
ATP	5957	3.512	16	292.16	0.00000024
citrate/isocitrate	311/5459771	5.077	13	0.00	0.106793758
citrate/isocitrate	311/5459771	4.555	15	43.86	0.106793758
dihydroorotate	5460289	18.581	3	60.08	1.46E-11
dihydroorotate	5460289	4.376	2	238.07	1.46E-11
dihydroxy-acetone phosphate	4643300	5.449	15	48.32	0.0000518
Sedoheptulose 7-phosphate	165007	4.473	2	203.32	2.93E-13
Sedoheptulose 7-phosphate	165007	3.922	16	170.08	2.93E-13
Sedoheptulose 7-phosphate	165007	3.274	15	45.65	2.93E-13
Sedoheptulose 7-phosphate	165007	3.243	13	40.33	2.93E-13
fructose-1,6-bisphosphate	5460765	5.104	15	48.32	0.0000207
glutamate	5460544	3.680	16	268.52	0.000000317
glutamate	5460544	3.356	2	220.12	0.000000317
glutathione†	124886	3.705	9	109.64	2.57E-09
glutathione disulfide†	975	3.545	2	228.12	N/A
hexose-phosphate	466/4459709	5.151	2	227.24	9.08E-11
hexose-phosphate	466/4459709	4.150	16	170.08	9.08E-11
hexose-phosphate	466/4459709	3.622	15	47.44	9.08E-11
hexose-phosphate	466/4459709	4.410	13	14.31	N/A
inosine	6021	3.825	14	113.37	2.44E-13
lactate	5460161	6.858	12	313.39	N/A
Lysine (possibly glutamine)	5962	3.518	15	5.25	N/A
N-acetyl-glucosamine-1-phosphate	25243937	5.662	2	214.65	1.43E-08
NAD+_posi	5892	4.114	2	219.24	0.0000846
NADP+_posi	15938972	3.672	5	132.89	0.0000846
orotate	967	18.649	5	78.88	3.43E-18
orotidine	160617	19.150	5	78.89	5.39E-18
orotidine-5'-phosphate	160617	18.740	5	78.89	9.25E-15
phenylpyruvate	997	3.629	2	207.77	1.49E-10
quinolinate	1066	4.348	13	201.64	2.12E-10
quinolinate	1066	3.448	15	279.00	2.12E-10
ribose-phosphate	77982/439236	3.677	12	171.77	1.02E-09
ribose-phosphate	77982/439236	3.608	2	206.89	1.02E-09
ribose-phosphate	77982/439236	3.423	16	259.79	1.02E-09
S-adenosyl homocysteine*	439155	3.701	8	98.70	3.70E-13
S-adenosyl homocysteine*	439155	5.414	8	92.85	1.63E-09
S-adenosyl-L-methionine	34755	7.064	8	101.89	0.00000021
serine	5951	3.504	16	206.14	4.16E-08
thiamine	1130	9.873	8	100.12	1.08E-10
threonine	6288	4.365	2	244.45	0.000000433
threonine	6288	3.586	5	36.32	0.000000433
UDP-D-glucose	8629	6.179	5	78.89	3.39E-10
UDP-D-glucose	8629	3.768	2	244.45	3.39E-10
UDP-N-acetyl-glucosamine	643981	5.685	2	219.24	0.0000113
UDP-N-acetyl-glucosamine	643981	3.665	5	78.89	0.0000113
valine	6287	11.957	3	60.07	6.65E-09
valine	6287	3.623	16	166.53	6.65E-09
valine	6287	3.246	12	231.79	6.65E-09

Sup. Table 1. Metabolites and their linkage LOD-scores. All 52 linkages are listed, sorted by metabolite name. Metabolites with multiple linkages are sorted by LOD-score. The chromosome and position of the closest marker are also given. For metabolites detected in both parental strains, the p-value of metabolite level differences between the parents is also shown. FDR of 5% corresponds to a p-value of 0.0898. * Same compound but in different ionization modes. †considered same compound
