# Supplementary material for: Genetic Basis of Metabolome Variation in Yeast
Source: PLoS Genet. 2014 Mar 6;10(3):e1004142. doi: 10.1371/journal.pgen.1004142 (PMC3945093; doi:10.1371/journal.pgen.1004142)
Supplement: Table S2 — Examining confidence intervals for pathway genes. Compounds are shown with the number of pathway genes and metabolic genes captured in their confidence intervals. Pathway genes for each compound are specified in the third column. For compounds with multiple linkages, metabolic gene number and pathway gene names are broken down by the chromosome of the linkage. Glutathione and glutathione-disulfide are combined, as are the positive and negative mode measurements for S-adenosyl-homocysteine. * While alcohol dehydrogenase (ADH1) is not specified as a gene in the same pathway as these metabolites, it is mentioned due to its role in glycolysis. Same as S-adenosyl-L-homocysteine-nega-1. (PDF) [file pgen.1004142.s007.pdf]

Compound	# Overlapping Pathway Genes	Overlapping Pathway Genes	# Metabolic Genes in CI(s)
aconitate	1	ACO1	16
ADP	8	PYC2, PGI1, ADH5, YPC1, MIS1, RIB7, ECM31, DUR1,2	38
allantoate	0		20
aspartate	1	II: PYC2	17 on Chr II 1 on Chr XVI
ATP-nega	6	MET16, ERG10, GLN1, MET12, ASN1, CIT3	27
citrate/isocitrate	0		1 on Chr XIII 4 on Chr XV
dihydroorotate	0		25 on Chr II 1 on Chr III
dihydroxy-acetone phosphate	0*	*ADH1	1
Sedoheptulose 7-phosphate	3	II: TKL2; XV: RKI1; XVI: TKL1	27 on Chr II 67 on Chr XIII 72 on Chr XV 17 on Chr XVI
fructose-1,6-bisphosphate	0*	*ADH1	1
glutamate	6	II: TYR1, LYS2, PYC2, ADH5; XVI: ARO7, GLN1	25 on Chr II 23 on Chr XVI
glutathione/glutathione-disulfide	1	II: GPX2	67 on Chr II 3 on Chr IX
hexose-phosphate	3	II: PYC2; XV: RKI1; XVI: TKL1	18 on Chr II 5 on Chr XIII 65 on Chr XV 17 on Chr XVI
inosine	2	ADE12, AAH1	14
lactate	0		2
lysine	6	CPA1, ADE2, CDC21, DFR1, HIS3, NPT1	72
N-acetyl-glucosamine-1-phosphate	0		16
NAD+	6	II: ADH5, PGI1, PYC2, MET8, PDB1, ARA1	21 on Chr II
NADP+	6	ALD5, CEM1, PRO3, ILV1, HOM3, ARG5,6	40
orotate	1	URA3	1
orotidi	1	URA3	1
orotidine-5'-phosphate	1	URA3	1
phenylpyruvate	1	II: ADH5	67 on Chr II
quinolinate	0		9 on Chr XIII 38 on Chr XV
ribose-phosphate	6	XII: ADE13, IMD3, PNP1, TAL1, URA4; XVI: TKL1	21 on Chr II 61 on Chr XII 58 on Chr XVI
S-adenosyl homocysteine	1	ERG7	22
S-adenosyl-L-methionine	0		4
serine	1	MET12	23
thiamine	0		2
threonine	3	V: GLY1, ILV1, UTR4	17 on Chr II 48 on Chr V
UDP-D-glucose	0		17 on Chr II 3 on Chr V
UDP-N-acetyl-glucosamine	1	V: PCM1	12 on Chr II 37 on Chr V
valine	5	III: ILV6; XII: ECM38, MEU1, PDC1, PDC5	2 on Chr III 60 on Chr XII 46 on Chr XVI

Sup. Table 2. Examining Confidence Intervals for Pathway Genes.
